# Definition, harms, and prevention of redundant systematic reviews

**DOI:** 10.1186/s13643-023-02191-8

**Published:** 2023-04-04

**Authors:** Livia Puljak, Hans Lund

**Affiliations:** 1 Center for Evidence-Based Medicine and Health Care, Catholic University of Croatia, Zagreb, Croatia; 2 Section Evidence-Based Practice, Western Norway University of Applied Sciences, Bergen, Norway

**Keywords:** Evidence synthesis, Redundancy, Replicability, Transparency, Duplication

## Abstract

**Background:**

Along with other types of research, it has been stated that the extent of redundancy in systematic reviews has reached epidemic proportions. However, it was also emphasized that not all duplication is bad, that replication in research is essential, and that it can help discover unfortunate behaviors of scientists. Thus, the question is how to define a redundant systematic review, the harmful consequences of such reviews, and what we could do to prevent the unnecessary amount of this redundancy.

**Main body:**

There is no consensus definition of a redundant systematic review. Also, it needs to be defined what amount of overlap between systematic reviews is acceptable and not considered a redundancy. One needs to be aware that it is possible that the authors did not intend to create a redundant systematic review. A new review on an existing topic, which is not an update, is likely justified only when it can be shown that the previous review was inadequate, for example, due to suboptimal methodology. Redundant meta-analyses could have scientific, ethical, and economic questions for researchers and publishers, and thus, they should be avoided, if possible. Potential solutions for preventing redundant reviews include the following: (1) mandatory prospective registration of systematic reviews; (2) editors and peer reviewers rejecting duplicate/redundant and inadequate reviews; (3) modifying the reporting checklists for systematic reviews; (4) developing methods for evidence-based research (EBR) monitoring; (5) defining systematic reviews; (6) defining the conclusiveness of systematic reviews; (7) exploring interventions for the adoption of methodological advances; (8) killing off zombie reviews (i.e., abandoned registered reviews); (9) better prevention of duplicate reviews at the point of registration; (10) developing living systematic reviews; and (11) education of researchers.

**Conclusions:**

Disproportionate redundancy of the same or very similar systematic reviews can lead to scientific, ethical, economic, and societal harms. While it is not realistic to expect that the creation of redundant systematic reviews can be completely prevented, some preventive measures could be tested and implemented to try to reduce the problem. Further methodological research and development in this field will be welcome.

## Background

Redundant means *unnecessary because it is more than is needed* [1]. For systematic reviews, it has been stated that the extent of their redundancy has reached “epidemic proportions” [2]. However, it was also emphasized that not all duplication is bad, that replication in research is essential, and that it can help discover unfortunate behaviors of scientists [3]. Thus, the question is how to define a redundant systematic review, the harmful consequences of such reviews, and what we could do to prevent the unnecessary amount of this redundancy.

## Definition of a systematic review

Before addressing the issue of defining redundant systematic review, it should be highlighted that we still do not even have a consensus definition of a systematic review. Multiple organizations and authors have provided definitions or characteristics of systematic reviews, but the analysis of those definitions and characteristics showed that they are heterogeneous and often vague in their defining characteristics [4]. For example, when a definition says that a systematic review should have a “systematic search,” without further details, it is unknown what precisely the “systematic” involves. Furthermore, it is unclear what constitutes a “clearly defined question and set of eligibility criteria,” etc. [4].

We might have our own ideas about what systematic search is. Then, there are methodological checklists such as AMSTAR 2 for systematic reviews of interventions, which define what a “comprehensive literature search” is. For item 4 in AMSTAR 2, asking, “Did the review authors use a comprehensive literature search strategy?,” the answer is partial yes if the authors have done all of the following: searching at least 2 databases (relevant to the research question), provided keyword and/or search strategy, and justified publication restrictions (e.g., language). For the answer yes, the systematic review should also have all of the following: searched the reference lists/bibliographies of included studies; searched trial/study registries; included/consulted content experts in the field, where relevant; searched for gray literature; and conducted a search within 24 months of completion of the review [5]. But, it is currently unclear whether there is a minimum threshold of this “comprehensive search” that should be a defining characteristic of a systematic review. The same holds for other methodological aspects of systematic reviews.

Thus, currently, we do not have a minimal consensus set of methodological criteria that would define a systematic review. Consequently, anything can be self-described as a “systematic review,” even though the review may not adhere to the minimal methodological expectations such as properly searching multiple sources.

## Definition of a redundant systematic review

There is no consensus definition of a redundant systematic review. David Moher offered some thoughts on how to define “more than is needed” for systematic reviews in 2013, suggesting that there is no magic number in the context of systematic reviews and meta-analysis regarding the “correct” amount of replication, and that [quote] “most people would probably consider that two or three systematic reviews on the same topic with similar eligibility criteria and outcomes is reasonable, whereas four or more would definitely be too many” [3].

Moher used expressions “duplication” and “similar” [3]. Other than the term redundant, other related terms used in the context of systematic reviews’ redundancy were “unnecessary” and “overlapping” [2]. However, it is unclear whether these terms should be used as synonyms. Something could be similar but not the same, and the extent of similarity that is acceptable to justify a new systematic review is unclear.

A lack of consensus definition of replication and reproduction in the context of systematic reviews was also highlighted recently [6]. One of the proposed definitions says that replication involves redoing the same study again to address the same question(s) of a prior study, while reproduction is reanalyzing the data collected in a prior study using the same data, computational steps, and analytic code as the original study [7].

However, there have been other takes on replication when it comes to systematic reviews. In 2020, Tugwell et al. proposed a consensus checklist that should help authors decide when to replicate systematic reviews of interventions [8]. The group acknowledged that there were no standards regarding the terminology and conceptual framework for the replication of systematic reviews. They proposed two distinct types of replication — direct and conceptual replication. Direct replication would be a purposeful repetition of the original research question for verification purposes. Conceptual replication would involve a purposeful revision of the original research question [8].

It was suggested [6] that only direct replication, as defined by Tugwell et al. [8], could be considered synonymous with duplication. However, when the authors decide to change the original question, it is not realistic to expect true replication, i.e., duplication [6]. Conceptual replication would, thus, result in a potential overlap with an original systematic review but not an exact duplication. It is unclear presently what amount of overlap is acceptable before being considered redundant.

For example, in 2021, Lunny et al. proposed a taxonomy for overlapping overviews of systematic reviews addressing health interventions [9]. The overlap was defined as a duplication of PICO (patients, interventions, comparators, outcomes) eligibility criteria and was not reported as an update or a replication. The overlapping overviews were classified into four categories — identical, nearly identical, partial, or subsumed [9]. Definitions for all four categories were proposed. For example, on one side of the spectrum, an identical overlap was defined as having a PICO identical to another overview, while a subsummation was defined as “when broad overviews subsumed the populations, interventions and at least one outcome of another overview” [9]. However, that taxonomy was proposed by one group of researchers from Canada, and it is unclear whether others in the global field of research methodology globally would agree with this definition. Furthermore, this taxonomy was proposed for a specific type of evidence synthesis — namely for overviews of systematic reviews addressing health interventions. It needs to be seen whether this taxonomy can be applied to systematic reviews that are not overviews. Also, it needs to be seen what amount of overlap is acceptable and not considered a redundancy. Furthermore, we would also need a definition of redundancy and overlap in evidence syntheses beyond those that are analyzing interventions.

## Intent to produce a redundant systematic review

When defining a direct and a conceptual replication of systematic reviews, Tugwell et al. used the word “purposeful,” indicating that the author team is embarking on replication on purpose [8]. Lunny et al. indicated that it should be considered whether the same team of authors or a team that had some authors in common has conducted an update of a previous overview. Alternatively, if those authors did not report that a review is an update, there is a possibility that a new evidence synthesis produced by the same/shared authors could be a form of self-plagiarism [9]. In the context of self-plagiarism, redundant/duplicate publication involves reporting identical or very similar data in two or more articles without explicitly stating that the data are being recycled [10].

However, multiple studies have explored the problem of overlapping systematic reviews that were published within a short period of time, and that did not have the same or shared author teams [11–14]. Thus, it could be hypothesized that many author teams are not embarking on producing a new systematic review with the purposeful intention of creating a redundant one.

There could be multiple reasons for an author team not being aware of an existing systematic review or protocol for a systematic review when starting a new one that will turn out to be overlapping. For example, perhaps the authors did not search the literature and registries such as PROSPERO, or they did not search it properly. Furthermore, it is possible that they could not find a protocol for a systematic review because the protocol was submitted to PROSPERO, but not yet published on the PROSPERO website at the time of the search. It has been reported that due to the increased volume of submitted registrations, PROSPERO had major delays in publishing those registrations, with some submissions waiting as long as 6 months to be published [15].

This has been rectified in the meantime by PROSPERO. According to the notice on the PROSPERO’s web site (accessed on October 28, 2022), submissions that pass a basic automated check and that are waiting for registration for more than 30 days are published automatically with a note that the PROSPERO team did not check those submissions [16]. With this change, chances of not finding duplicate reviews because they are in the PROSPERO system waiting to be published is very much reduced.

A recent study examined the extent and nature of duplication in a sample of PROSPERO registration records related to COVID-19. Of 1054 registration records examined across 4 of the categories used by authors to classify their COVID-19 review protocols, 13% were identified as having been submitted when at least one very similar protocol was already registered in PROSPERO. Over half of these did not acknowledge any similar existing reviews during pre-registration screening questions, less than a quarter acknowledged similar existing reviews in their publicly viewable registration record, and only 4 recorded a reason for replicating, despite being advised during the registration process that the reasons for duplication should be made clear (*personal communication; study results under consideration in a journal*).

Beyond PROSPERO, there are other sources where one can register a systematic review [17], and the authors can publish a systematic review protocol as a journal article [18]. Thus, the question is how comprehensive the search should be and what sources to search when preparing for a new systematic review.

Also, it is possible that an author team had done their due diligence when they started considering a new systematic review, and that there was indeed not an identical or similar review or a registration/protocol of such a review available, but that this author team took a long time to produce a systematic review, and then, somebody else conducted the same or similar review in the meantime. It is known that some reviews take a very long time from protocol to registration. Some Cochrane reviews took more than 8 years from protocol publication to a publication of a full review [19]. It is also known that there are so-called zombie reviews, which were registered, but then abandoned by their author team [20]. Multiple studies have shown that many PROSPERO records are not published as a full systematic review for a long time [20, 21]. And while more systematic reviews are registered in PROSPERO each year, few of them have an up-to-date status [22].

Another issue in relation to the registration of systematic reviews is that potential author teams may identify very similar already-registered systematic reviews, but they do not know when, or even if, that registered review will be completed and made publicly available unless they contact the authors. In the meantime, authors may decide to go ahead and conduct their own review.

## When is a redundant systematic review justified?

If the author team is aware of the existing completed systematic review, the only case when a duplicate, directly replicated systematic review could be justified is if the first review used inadequate methods. In that case, the other team can decide to address that topic all over again with appropriate methods. Such cases should be adequately justified in the new review by supporting this decision with evidence that the previous review was inadequate. For example, the new team could provide a transparent analysis of the methodological quality of the first review using the AMSTAR 2 tool [5], with details of this analysis reported in a supplementary file.

Otherwise, if the author team is aware of the existing completed systematic review with adequate methods, the only proper decision to move forward with the same topic would be to conduct an update of that systematic review. Also, in this case, it would be ideal first to check whether the original author team is planning to conduct an update.

Thus, a redundant review is never justified unless it is clearly defined as an intended duplication, replication, or update. But proper justification should be provided in all those cases. For example, updated versions of systematic reviews could also contribute to redundancy. Some updates may not be considered justified. For example, if an existing large review is updated with one new study that is unlikely to impact results, it is reasonable to question the purpose of such an update.

Cochrane has tried to address this by stabilizing certain systematic reviews. Cochrane reviews could be designated “stable” if they were considered as not needing to be updated but highly likely to be current. An analysis of stabilized reviews conducted by Babic et al. indicated that the most common reason for stabilizing a Cochrane review was “last search did not identify any potentially relevant studies likely to change conclusions” [23]. That means that even if the latest search finds new relevant evidence, it can be considered that the new evidence is not sufficiently substantive to warrant an update. Even though currently it is unclear when is a systematic review conclusive and should not be updated any more [24], the authors should be very cautious when deciding whether an update of a systematic review is warranted.

## Harms caused by redundant reviews

Chapelle et al. suggested that the multiplication of redundant meta-analyses raises scientific, ethical, and economic questions for researchers and publishers [25]. The scientific harm of redundant reviews can be seen as an accumulation of avoidable research waste.

Knowingly engaging in wasteful research practices can also be seen as unethical behavior. It is also possible that someone could purposefully produce a biased systematic review to use it for their own vested interests — this would also be unethical [26].

In the context of clinical trials, it has been suggested that a trial can be considered wasteful if it does not deliver new knowledge that can justify risks taken by study participants, the effort of researchers, and the allocated financial and other resources [27]. While systematic reviews do not involve human participants, unnecessary redundancy and overlap still lead to wasted effort and resources, i.e., economic harm. The authors could have used the allocated human, financial, and other resources to produce research that is needed instead.

One of the harms associated with redundant systematic reviews could be mistrust of this type of evidence. This could be considered societal harm. The warning about the “mass production of redundant, misleading, and conflicted systematic reviews and meta-analyses” [2] has even been linked with the concerns that systematic reviews and meta-analyses are not useful research [28]. Chevret et al. dramatically declared that “a veritable tsunami” of systematic reviews and meta-analyses “will drown clinicians, public health officials and investigators in perpetuity,” describing the situation with systematic reviews as “an ideological, publication-fuelled bubble which, similar to the alchemists of centuries ago, promises to make gold out of clods of earth” [28]. However, while acknowledging that with systematic reviews there is a high risk of redundancy, flawed methodology, conflict of interest-driven biases, and misinterpretation of evidence, Annane et al. concluded that there is simply no alternative to using systematic reviews [29]. While some systematic reviews may be flawed and redundant, many rigorously conducted systematic reviews still enable informed decision-making in health [29].

If we put aside these concerns, it could also be argued that redundant systematic reviews, if they are conducted adequately and if they have the same results and conclusions, will not lead to any harm in terms of ill-informed decisions regarding human health. However, there is a documented problem that multiple author teams are producing systematic reviews on the same topic but with discordant results [12, 25, 30]. The availability of discordant reviews can lead to cherry-picking of systematic reviews that will fit someone’s conscious or unconscious agenda.

Devoid of a hidden agenda, the question is how the readers should choose which systematic review to trust when faced with discordant systematic reviews. A survey of methodologists and clinicians who were presented with evidence from four overlapping and discordant systematic reviews and meta-analyses showed that the preferred approach was to use only high-quality systematic reviews [31].

## Measures for preventing redundant systematic reviews

It is not realistic to expect that the problem of truly redundant systematic reviews will disappear completely. However, some preventive measures could potentially alleviate the extent of the redundancy.

### Mandatory prospective registration of systematic reviews

In 2005, the International Committee of Medical Journal Editors (ICMJE) instituted mandatory prospective registration of clinical trials [32]. Editors, as gatekeepers, could impose the prospective registration request for systematic reviews. Among the sample of systematic reviews published in 2018, only 32% reported they were registered in PROSPERO [22]. A study of 357 systematic reviews about human interventions published in January 2020 and January 2021 showed that of the non-Cochrane reviews, 135 (38%) reviews had a protocol either registered, published, or both. PROSPERO was dominantly used for protocol registration (*n* = 129; 96%) [33]. Only 25% of systematic reviews on COVID-19 treatments were registered in PROSPERO [34].

With a larger proportion of systematic reviews preregistered, there could be higher chances that redundant systematic reviews could be avoided. This is a hypothesis that remains to be confirmed, but parallels can be drawn with trial registration, for which it was written “prospective clinical trial registration: not sufficient, but always necessary” [35]. While it may be difficult to estimate whether trial registration has contributed to less duplicate/redundant trials, it has been highlighted that trial registration can prevent “sins of omission or commission,” such as not reporting nonsignificant results or changing the pre-specified primary outcome [35].

### Editors and peer reviewers rejecting duplicate/redundant and inadequate reviews

Some scholarly journals prize novelty, while others indicate in their instructions for authors that they accept scientifically rigorous research, regardless of novelty. While it has already been emphasized that replication in science can be welcome, editors and peer reviewers of journals that are not focused on novelty could opt to reject duplicate systematic reviews.

Journals should also refuse to publish systematic reviews not meeting rigorous standards [29]. However, since there is plenty of evidence that less than rigorous systematic reviews are still being published, interventions are needed to ensure better checks of submitted systematic reviews.

In 2022, Berlin et al. published an editorial addressing editors’ perspectives regarding the assessment of systematic reviews and meta-analyses [36]. Even though the editors of JAMA Network Open did not establish explicit standards for determining the acceptability of systematic reviews/meta-analyses, they implicitly developed criteria for judging such submissions. They ask authors to answer a series of questions in a cover letter, including whether the new article is necessary, whether the such article was published within the past five years, and whether it addresses a secondary question, is it unique, and does it adequately address the heterogeneity and diversity of the populations studied among the included studies. When authors do not address those questions, editors of the JAMA Network Open may return the manuscript and ask them to do so before further consideration [36]. However, even if authors provide all those answers, this does not mean that their answers will be justified or in line with the published evidence. So, even though this intervention is trying to put the onus back on the authors, and rightfully so, editors and peer reviewers will still need to be vigilant about verifying the authors’ claims about the need for a new systematic review.

### Modifying the reporting checklists for systematic reviews

Item 3 of PRISMA 2020 instructs authors to provide a rationale for the systematic reviews: “Describe the rationale for the review in the context of existing knowledge” [37]. Researchers who will read only this checklist without reading the accompanying explanation and elaboration might be misled into thinking that citing any existing knowledge is sufficient. If the researchers decide to read the explanation and elaboration, they will see that it is considered an essential element for this item to check if other systematic reviews are addressing the same or a largely similar question. However, there is no recommendation about how to check whether there are other such systematic reviews. For example, this essential element could instruct researchers to search multiple databases for this purpose. Additionally, the explanation and elaboration for this item do not mention the search of registries such as PROSPERO.

Item 6 of the PRISMA-P for protocols, which was published in 2015, also says “Describe the rationale for the review in the context of what is already known,” but the full manuscript describing the PRISMA-P does not urge the researchers to search the registrations [38].

So, the PRISMA and PRISMA-P item could be revised from “Describe the rationale for the review in the context of existing knowledge” into “Describe the rationale for the review in the context of existing knowledge, including whether other systematic reviews are addressing the same or a largely similar question.”

When authors are prompted in this way to look for existing systematic reviews, they could be faced with multiple systematic reviews that could potentially have discordant results [12]. A recent publication explored how methodologists and clinicians make a decision when faced discordant evidence from multiple systematic reviews formalized in structured tables [31]. Further work is needed to create practical guidance on making sense of multiple reviews and what might constitute redundancy.

### Developing methods for evidence-based research (EBR) monitoring

EBR involves the use of existing research systematically and transparently to inform the need for a new study [39]. However, it has been recognized that there are limited methods for monitoring the EBR implementation. One such method is citation analysis, which involves studying the frequency, patterns, and graphs of citations [40]. For example, one could study citations in a systematic review to see whether the prior systematic reviews were properly acknowledged and the need for a new one adequately justified. In 2022, Nørgaard et al. published a report about the analysis of 27 studies that used citation analysis, but the methodological approaches to citation analysis were very heterogeneous [41]. Further methodological work in the field of EBR monitoring may yield solutions that would help create alerts about redundancies and help prevent further redundant studies.

### Defining systematic reviews

If we could adopt a clear, detailed consensus definition of a systematic review with minimal methodological standards that a systematic review should adhere to [4], we could focus our efforts and considerations only on studies that meet those criteria. Then, it could become apparent that many “redundant systematic reviews” are actually not systematic reviews at all.

### Defining the conclusiveness of systematic reviews

Currently, we do not have a definition of conclusive research, i.e., a method to declare that we have had enough/sufficient evidence, and that new research on a topic is no longer needed [23, 24, 42]. If we could define that, we could declare that some research topics need to be closed, and that it is not necessary to conduct further research on this topic, primary or secondary.

The practical question could be who would oversee the process of “closing” conclusive systematic reviews. For example, a registry of closed systematic review topics could be created. But before we can ask who would oversee the process, we would need to define the criteria for considering a review conclusive. Then, part of the regular systematic review methodology would be the assessment of the conclusiveness of evidence, and each systematic review could report at the end of the results whether the results are conclusive against those criteria or not.

### Exploring interventions for the adoption of methodological advances

If we consider that a new systematic review, representing a direct replication, is needed because the previously published review had inadequate methods, then this is a call to further address those methodological inadequacies. We have a reporting guideline for systematic reviews and its extensions [43], as well as Cochrane’s methodological expectations for systematic reviews of interventions [44], Joanna Briggs Institute (JBI) Checklist for Systematic Reviews [45], AMSTAR 2 tool for assessing methodological quality [5], a ROBIS tool for assessing the risk of bias in systematic reviews [46], etc. However, despite the existence of those tools, it has been reported in multiple studies that published systematic reviews do not adhere to guidance from those tools [47–52]. Thus, further studies in the field of research methodology should explore interventions that would improve how systematic reviews are planned, conducted, and reported. If we could ensure the production of more high-quality systematic reviews, there would be less need to replicate them due to inadequate quality.

### Killing off zombie reviews

If we could remove zombie reviews [20], i.e., abandoned reviews, from a registry such as PROSPERO, we could potentially prevent some apparent duplication, and some author teams could embark on a new systematic review without being scared off by zombies. Olson and Basil have analyzed evidence from literature about interventions for killing zombies. They concluded that the best evidence-based practice would be to kill a zombie with a skull-penetration intervention (class of evidence, level 2B; strength of recommendation, level C) [53]. For zombie systematic reviews, other interventions would be needed. Andrade et al. have suggested measures for fighting back zombie reviews in PROSPERO [20]. These suggestions include developing a system that will help with keeping the up-to-date status of a systematic review registration by sending annual emails that need to be responded with a certain period of time. Otherwise, the nonresponse will be flagged on the registration [20]. A more labor-intensive suggestion was to hire staff that will search for PROSPERO unique identifiers in the published literature, to update the PROSPERO record status [20]. This literature search could also potentially be solved with automation.

PROSPERO is currently developing a new version, which will enhance the functionality of sending reminders to authors, requesting that they update their review status and add publication details. The dates of registration and planned review completion are available in PROSPERO records. So, if those planning a new review find a suspect registered “zombie” review that addresses their research question, but looks dormant, they can contact the author directly to ask whether it will be completed. With over 170,000 records in PROSPERO, it would currently not be feasible or affordable to have PROSPERO staff check for associated publications (*personal communication*).

### Better prevention of duplicate reviews at the point of registration

Automated tools could be developed that would search the registrations and literature when a new systematic review registration is submitted to the registry. Such a tool could search for similarities in PICO and alert the submitter about the similarity check results.

Of course, even if this functionality is implemented in the registries, the responsibility would still be on the author teams to provide information on existing systematic reviews that address the same or similar question and for the authors to justify why a new systematic review is warranted. System’s approaches can facilitate this, but the responsibility ultimately lies with the authors to avoid or explain duplication.

### Developing living systematic reviews

To counter the epidemic proportions of redundant systematic reviews, Chapelle et al. suggested developing living systematic reviews [25]. While this idea is worthwhile, it is likely unrealistic to develop such living evidence syntheses for all areas of research. This would require massive resources, and it would be very difficult to ensure the sustainability of a myriad of living systematic reviews.

Furthermore, we would need a system that would coordinate the living systematic reviews, and the issue of their ownership/authorship would need to be addressed. Without some form of centralized system of managing living systematic reviews, some duplication would be inevitable.

### Education of researchers

Curricula for education about methods for developing systematic reviews should address the problem of redundant systematic reviews. Researchers who want to conduct new reviews need to be educated on searching for existing ones in registries and databases that publish completed studies. Also, when researchers find the same or similar systematic review already registered, they should not instantly give up on that topic. Instead, it is advisable to check with the authors whether this review is still ongoing or it has turned into a zombie.

## Conclusions

In science, redundancy can be seen as a feature of the system, not a bug [54]. However, disproportionate redundancy of the same or very similar systematic reviews can lead to scientific, ethical, economic, and societal harms. While it is not realistic to expect that the creation of redundant systematic reviews can be completely prevented, some preventive measures could be tested and implemented to try to reduce the problem. Preventive measures that could be accomplished quickly with few resources include revision of the PRISMA and PRISMA-P item regarding the existing evidence, designing uniform list of questions that authors would need to address in a cover letter upon submission, and instituting a mandatory registration of systematic review protocols. For other preventive measures, further methodological research and development in this field will be welcome.

## Data Availability

Not applicable. This is a commentary, and we did not generate any data and materials during the writing of this commentary.

## Abbreviations

EBR: Evidence-based research
ICMJE: International Committee of Medical Journal Editors
JBI: Joanna Briggs Institute
PICO: Patients, interventions, comparators, outcomes

## References

[1] Redundant. Cambridge Dictionary. Available at: https://dictionary.cambridge.org/dictionary/english/redundant.

[2] Ioannidis JP (2016). The mass production of redundant, misleading, and conflicted systematic reviews and meta-analyses. Milbank Q.

[3] Moher D (2013). The problem of duplicate systematic reviews. BMJ.

[4] Krnic Martinic M, Pieper D, Glatt A, Puljak L (2019). Definition of a systematic review used in overviews of systematic reviews, meta-epidemiological studies and textbooks. BMC Med Res Methodol.

[5] Shea BJ, Reeves BC, Wells G, Thuku M, Hamel C, Moran J, Moher D, Tugwell P, Welch V, Kristjansson E (2017). AMSTAR 2: a critical appraisal tool for systematic reviews that include randomised or non-randomised studies of healthcare interventions, or both. BMJ.

[6] Puljak L, Pieper D (2022). Replicability in the context of systematic reviews: a call for a framework with considerations regarding duplication, overlap, and intentionality. J Clin Epidemiol.

[7] Page MJ, Moher D, Fidler FM, Higgins JPT, Brennan SE, Haddaway NR, Hamilton DG, Kanukula R, Karunananthan S, Maxwell LJ (2021). The REPRISE project: protocol for an evaluation of REProducibility and Replicability In Syntheses of Evidence. Syst Rev.

[8] Tugwell P, Welch VA, Karunananthan S, Maxwell LJ, Akl EA, Avey MT, Bhutta ZA, Brouwers MC, Clark JP, Cook S (2020). When to replicate systematic reviews of interventions: consensus checklist. BMJ.

[9] Lunny C, Reid EK, Neelakant T, Chen A, Zhang JH, Shinger G, Stevens A, Tasnim S, Sadeghipouya S, Adams S (2022). A new taxonomy was developed for overlap across ‘overviews of systematic reviews’: a meta-research study of research waste. Res Synth Methods.

[10] Ding D, Nguyen B, Gebel K, Bauman A, Bero L (2020). Duplicate and salami publication: a prevalence study of journal policies. Int J Epidemiol.

[11] Augustin G, Boric M, Barcot O, Puljak L (2020). Discordant outcomes of laparoscopic versus open appendectomy for suspected appendicitis during pregnancy in published meta-analyses: an overview of systematic reviews. Surg Endosc.

[12] Riva N, Puljak L, Moja L, Ageno W, Schunemann H, Magrini N, Squizzato A (2018). Multiple overlapping systematic reviews facilitate the origin of disputes: the case of thrombolytic therapy for pulmonary embolism. J Clin Epidemiol.

[13] Perez-Gaxiola G, Verdugo-Paiva F, Rada G, Florez ID (2021). Assessment of duplicate evidence in systematic reviews of imaging findings of children with COVID-19. JAMA Netw Open.

[14] McDonald S, Turner S, Page MJ, Turner T (2022). Most published systematic reviews of remdesivir for COVID-19 were redundant and lacked currency. J Clin Epidemiol.

[15] Puljak L (2021). Delays in publishing systematic review registrations in PROSPERO are hindering transparency and may lead to research waste. BMJ Evid Based Med.

[16] PROSPERO. Important notice. Available at: https://www.crd.york.ac.uk/prospero/.

[17] Pieper D, Rombey T (2022). Where to prospectively register a systematic review. Syst Rev.

[18] Rombey T, Puljak L, Allers K, Ruano J, Pieper D (2020). Inconsistent views among systematic review authors toward publishing protocols as peer-reviewed articles: an international survey. J Clin Epidemiol.

[19] Runjic E, Behmen D, Pieper D, Mathes T, Tricco AC, Moher D, Puljak L (2019). Following Cochrane review protocols to completion 10 years later: a retrospective cohort study and author survey. J Clin Epidemiol.

[20] Andrade R, Pereira R, Weir A, Ardern CL, Espregueira-Mendes J (2019). Zombie reviews taking over the PROSPERO systematic review registry. It’s time to fight back!. Br J Sports Med.

[21] Runjic E, Rombey T, Pieper D, Puljak L (2019). Half of systematic reviews about pain registered in PROSPERO were not published and the majority had inaccurate status. J Clin Epidemiol.

[22] Rombey T, Doni K, Hoffmann F, Pieper D, Allers K (2020). More systematic reviews were registered in PROSPERO each year, but few records’ status was up-to-date. J Clin Epidemiol.

[23] Babic A, Poklepovic Pericic T, Pieper D, Puljak L (2020). How to decide whether a systematic review is stable and not in need of updating: analysis of Cochrane reviews. Res Synth Methods.

[24] Babic A, Poklepovic Pericic T, Pieper D, Puljak L (2022). When is the evidence conclusive? Analysis of systematic reviews for which Cochrane declared that conclusions will not change with further studies. Res Synth Methods.

[25] Chapelle C, Ollier E, Girard P, Frere C, Mismetti P, Cucherat M, Laporte S (2021). An epidemic of redundant meta-analyses. J Thromb Haemost.

[26] Evans I, Thornton H, Chalmers I, Glasziou P (2011). Recognizing vested interests and spin in systematic reviews. Testing Treatments, 2nd Edition.

[27] Kim D, Hasford J (2020). Redundant trials can be prevented, if the EU clinical trial regulation is applied duly. BMC Med Ethics.

[28] Chevret S, Ferguson ND, Bellomo R (2018). Are systematic reviews and meta-analyses still useful research? No. Intensive Care Med.

[29] Annane D, Jaeschke R, Guyatt G (2018). Are systematic reviews and meta-analyses still useful research? Yes. Intensive Care Med.

[30] Jadad AR, Cook DJ, Browman GP (1997). A guide to interpreting discordant systematic reviews. CMAJ.

[31] Puljak L, Parmelli E, Capobussi M, Gonzalez-Lorenzo M, Squizzato A, Moja L, Riva N (2022). Mitigating disputes originated by multiple discordant systematic reviews and meta-analyses: a survey of methodologists and clinicians. Front Res Metr Anal.

[32] De Angelis C, Drazen JM, Frizelle FA, Haug C, Hoey J, Horton R, Kotzin S, Laine C, Marusic A, Overbeke AJ (2004). Clinical trial registration: a statement from the International Committee of Medical Journal Editors. Lancet.

[33] van der Braak K, Ghannad M, Orelio C, Heus P, Damen JAA, Spijker R, Robinson K, Lund H, Hooft L (2022). The score after 10 years of registration of systematic review protocols. Syst Rev.

[34] Siemens W, Nothacker J, Stadelmaier J, Meerpohl JJ, Schmucker C (2022). Three out of four published systematic reviews on COVID-19 treatments were not registered and one-third of those registered were published: a meta-research study. J Clin Epidemiol.

[35] Smith SM, Dworkin RH (2018). Prospective clinical trial registration: not sufficient, but always necessary. Anaesthesia.

[36] Berlin JA, Rubenfeld GD, O’Cearbhaill RE, Shah AS, Fihn SD (2022). Keeping meta-analyses fresh. JAMA Netw Open.

[37] Page MJ, McKenzie JE, Bossuyt PM, Boutron I, Hoffmann TC, Mulrow CD, Shamseer L, Tetzlaff JM, Akl EA, Brennan SE (2021). The PRISMA 2020 statement: an updated guideline for reporting systematic reviews. BMJ.

[38] Shamseer L, Moher D, Clarke M, Ghersi D, Liberati A, Petticrew M, Shekelle P, Stewart LA, Group P-P (2015). Preferred reporting items for systematic review and meta-analysis protocols (PRISMA-P) 2015: elaboration and explanation. BMJ.

[39] Robinson KA, Brunnhuber K, Ciliska D, Juhl CB, Christensen R, Lund H, Evidence-Based Research N (2021). Evidence-based research series-paper 1: what evidence-based research is and why is it important?. J Clin Epidemiol.

[40] van Wesel M (2016). Evaluation by citation: trends in publication behavior, evaluation criteria, and the strive for high impact publications. Sci Eng Ethics.

[41] Norgaard B, Briel M, Chrysostomou S, Ristic Medic D, Buttigieg SC, Kiisk E, Puljak L, Bala M, Pericic TP, Lesniak W (2022). A systematic review of meta-research studies finds substantial methodological heterogeneity in citation analyses to monitor evidence-based research. J Clin Epidemiol.

[42] Bastian H, Hemkens LG. Enough evidence and other endings: a descriptive study of stable Cochrane systematic reviews in 2019. medRxiv. 2019:19013912. https://www.medrxiv.org/content/10.1101/19013912v1

[43] Page MJ, McKenzie JE, Bossuyt PM, Boutron I, Hoffmann TC, Mulrow CD, Shamseer L, Tetzlaff JM, Akl EA, Brennan SE (2021). The PRISMA 2020 statement: an updated guideline for reporting systematic reviews. J Clin Epidemiol.

[44] Higgins JPT, Lasserson T, Chandler J, Tovey D, Thomas J, Flemyng E, Churchill R (2022). Methodological Expectations of Cochrane Intervention Reviews.

[45] Checklist for systematic reviews. Available at:https://jbi.global/critical-appraisal-tools

[46] Whiting P, Savovic J, Higgins JP, Caldwell DM, Reeves BC, Shea B, Davies P, Kleijnen J, Churchill R, group R (2016). ROBIS: a new tool to assess risk of bias in systematic reviews was developed. J Clin Epidemiol.

[47] Rombey T, Lochner V, Puljak L, Konsgen N, Mathes T, Pieper D (2020). Epidemiology and reporting characteristics of non-Cochrane updates of systematic reviews: a cross-sectional study. Res Synth Methods.

[48] Dosenovic S, Jelicic Kadic A, Vucic K, Markovina N, Pieper D, Puljak L (2018). Comparison of methodological quality rating of systematic reviews on neuropathic pain using AMSTAR and R-AMSTAR. BMC Med Res Methodol.

[49] Maticic K, Krnic Martinic M, Puljak L (2019). Assessment of reporting quality of abstracts of systematic reviews with meta-analysis using PRISMA-A and discordance in assessments between raters without prior experience. BMC Med Res Methodol.

[50] Garcia-Alamino JM, Lopez-Cano M, Kroese L, Helgstrand F, Muysoms F (2019). Quality assessment and risk of bias of systematic reviews of prophylactic mesh for parastomal hernia prevention using AMSTAR and ROBIS tools. World J Surg.

[51] Matthias K, Rissling O, Pieper D, Morche J, Nocon M, Jacobs A, Wegewitz U, Schirm J, Lorenz RC (2020). The methodological quality of systematic reviews on the treatment of adult major depression needs improvement according to AMSTAR 2: a cross-sectional study. Heliyon.

[52] Pieper D, Hellbrecht I, Zhao L, Baur C, Pick G, Schneider S, Harder T, Young K, Tricco AC, Westhaver E (2022). Impact of industry sponsorship on the quality of systematic reviews of vaccines: a cross-sectional analysis of studies published from 2016 to 2019. Syst Rev.

[53] Olson DM, Bazil JC (2019). Of zombies and evidence. J Neurosci Nurs.

[54] Crotty D (2019). The value of redundancy in research, or, in research, redundancy has value. The Scholarly Kitchen.

